# Supplementary material for: Overexpression of a Malus baccata (L.) Borkh WRKY Factor Gene MbWRKY33 Increased High Salinity Stress Tolerance in Arabidopsis thaliana
Source: Int J Mol Sci. 2025 Jun 18;26(12):5833. doi: 10.3390/ijms26125833 (PMC12193393; doi:10.3390/ijms26125833)
Supplement: Supplementary file 1 [file ijms-26-05833-s001.zip › Supplementary Figure.pdf]

```
1       ATGACTTCTTCCTTCACTCACCTCCTCACAAGCAACATGGAGAACAGCGGGATTGGACAGGACAGGACTAATTGGGGGGGACTATTCTCG
1        M  T  S  S  F  T  H  L  L  T  S  N  M  E  N  S  G  I  G  Q  D  R  T  N  W  G  G  L  F  S
91      GACTATTCTTCTAATCCGAACCGGTTCACGGACAGAAACGGGACTGATATCCCCAAGTTCAAGTCACTCCAGCCTCCTTCTCTGCCTCTC
31       D  Y  S  S  N  P  N  R  F  T  D  R  N  G  T  D  I  P  K  F  K  S  L  Q  P  P  S  L  P  L
181     TCTCCTCCCCCTGTTTCCCCTTCTTCTTACTTGACTTCCACGCCGGCTTTTAGTCCCACCGATTTTCTCAGCTCACCCATGTTCCTCTCT
61       S  P  P  P  V  S  P  S  S  Y  L  T  S  T  P  A  F  S  P  T  D  F  L  S  S  P  M  F  L  S
271     TCCTCATATAATTTTGAATCTCCAACAACAGGAGCTTTTTCGAGTCAGGTTTTCGATTGGATGAATAATACTAAAGACACCCAGCAAGGA
91       S  S  Y  N  F  E  S  P  T  T  G  A  F  S  S  Q  V  F  D  W  M  N  N  T  K  D  T  Q  Q  G
361     ATTAAGGATAAAGAGGAGCCGAAATTGTTCTCCGATTTCTCGTTCCAACCGGAATCGAGGCCTGCTACTAATCTTCAATCTTCTTCTAGC
121      I  K  D  K  E  E  P  K  L  F  S  D  F  S  F  Q  P  E  S  R  P  A  T  N  L  Q  S  S  S  S
451     ATGGTTTCAGTGGAAGAACCATTCAAAGGAGAAAGGAAATCATGGGATTTTAGTGCTGAGAAGACGGGAGTGAAGTCCGAATTCGAGCCA
151      M  V  S  V  E  E  P  F  K  G  E  R  K  S  W  D  F  S  A  E  K  T  G  V  K  S  E  F  E  P
541     ATTGAAGCGAATACGCAGAGTAATGGACTTAATGGTGCTCCAAAGTCCGATTACTTGCATTCTAATCAATGTTCGCAATATGCTCGAGAA
181      I  E  A  N  T  Q  S  N  G  L  N  G  A  P  K  S  D  Y  L  H  S  N  Q  C  S  Q  Y  A  R  E
631     CAAAAATCGGATGATGGCTTCAATTGGAGGAAGTATGGGCAGAAACAAGTGAAGGGTAGCGAAAATCCGCGGAGTTATTACAAGTGCACG
211      Q  K  S  D  D  G  F  N  W  R  K  Y  G  Q  K  Q  V  K  G  S  E  N  P  R  S  Y  Y  K  C  T
721     TTTCCAAATTGCCCCACAAAGAAGAAGGTTGAGAGATCATTGGACGGACAGATTACTCAAATTGTGTACAAGGGTAATCACAACCATCCT
241      F  P  N  C  P  T  K  K  K  V  E  R  S  L  D  G  Q  I  T  Q  I  V  Y  K  G  N  H  N  H  P
811     AAGCCTCAGTCCACAAGAAGATCAAGCTCCAACTCGATTCAGGGTTCTTTCTATGGAATCTCTGATCAATCCGTTCCGACGTTATCCAAT
271      K  P  Q  S  T  R  R  S  S  S  N  S  I  Q  G  S  F  Y  G  I  S  D  Q  S  V  P  T  L  S  N
901     CCGAAAGTTGAGTCCGTCTCACTACAGGAGGATTCTTCTACCTCAATTGGAGAGGATGAGTTTGAACAAAACTCTCCGATAAGTAATTCA
301      P  K  V  E  S  V  S  L  Q  E  D  S  S  T  S  I  G  E  D  E  F  E  Q  N  S  P  I  S  N  S
991     GTAGGAGCTGAAGATGAAAACGAACCTGAGGCGAAAAGATGGAAGGGAGACAATGCAAATGATCAGTCATATGCATCTTCTGGCGGTAGA
331      V  G  A  E  D  E  N  E  P  E  A  K  R  W  K  G  D  N  A  N  D  Q  S  Y  A  S  S  G  G  R
1081    ATTGTGAAAGAACCGAGAATTGTAGTGCAGACGACGAGCGAAATCGATATTCTGGATGACGGGTATAGGTGGAGGAAATATGGACAGAAA
361      I  V  K  E  P  R  I  V  V  Q  T  T  S  E  I  D  I  L  D  D  G  Y  R  W  R  K  Y  G  Q  K
1171    GTAGTGAAGGGAAATCCAAATCCAAGGAGCTACTACAAATGCACGTCCGTAGGTTGTCCAGTGAGGAAGCATGTGGAAAGAGCATCGCAG
391      V  V  K  G  N  P  N  P  R  S  Y  Y  K  C  T  S  V  G  C  P  V  R  K  H  V  E  R  A  S  Q
1261    GACACAAGGGCGGTGATCACCACGTACGAAGGGAAGCACAACCACGATGTTCCGGCAGCGCGAGGCAGCGGGAATTATAGCAATGCAAGT
421      D  T  R  A  V  I  T  T  Y  E  G  K  H  N  H  D  V  P  A  A  R  G  S  G  N  Y  S  N  A  S
1351    AGACCTGCTGCCGATAATAACAGCAACAATGTGTCCATGGCTGTGAGGCCCTTGGCATTGCCTAACCATTCTAATTTGAGCTACCTCAAC
451      R  P  A  A  D  N  N  S  N  N  V  S  M  A  V  R  P  L  A  L  P  N  H  S  N  L  S  Y  L  N
1441    TCTCTTCAGAACGCAAGGCAGCCAGCAACCACTGAAAGCCAACCGCCGTATACACTCAAAATGCAGAGTGCAGGAAGTTATGGATTTTCT
481      S  L  Q  N  A  R  Q  P  A  T  T  E  S  Q  P  P  Y  T  L  K  M  Q  S  A  G  S  Y  G  F  S
1531    GAGTTCTGA
511      E  F  *
```

Figure S1. Nucleotide and deduced amino acid sequence of *MbWRKY33*. Black underlining indicates the start codon and the stop codon, yellow and red underlining indicate the two conserved structural domains, respectively. The yellow boxes and red squares represent the C2H2-type zinc finger structures of Class I A WRKY transcription factors. * stands for termination codon
