# Supplementary material for: Overexpression of a Malus baccata (L.) Borkh WRKY Factor Gene MbWRKY33 Increased High Salinity Stress Tolerance in Arabidopsis thaliana
Source: Int J Mol Sci. 2025 Jun 18;26(12):5833. doi: 10.3390/ijms26125833 (PMC12193393; doi:10.3390/ijms26125833)
Supplement: Supplementary file 1 [file ijms-26-05833-s001.zip › SupplementaryTable.pdf]

Supplementary Materials

Supplementary Table S1. List of primers used in this study.

**Primer**	**Sequence (5-3')**	**Purpose**
MbWRKY33-F	ATGACTTCTTCCTTCACTCACCTCC	full-length cDNA of *MbWRKY33*
MbWRKY33-R	TCAGAACTCAGAAAATCCATAACTTCC	full-length cDNA of *MbWRKY33*
MbWRKY33-sl F	GACGTCTTCGAGCTCGGTACCATGACTTCTTCCTTCACTCACCTCC	For subcellular localization
MbWRKY33-sl R	CATGTCGACTCTAGAGGATCCTCAGAACTCAGAAAATCCATAACTTCC	For subcellular localization
MbWRKY33-qF	ACCTGAGGCGAAAAGATGGA	qPCR
MbWRKY33-qR	CGGTTGGCTTTCAGTGGTTG	qPCR
MbActin-F	ACACGGGGAGGTAGTGACAA	qPCR
MbActin-R	CCTCCAATGGATCCTCGTTA	qPCR
Atactin-F	CTTGCACCAAGCAGCATGAA	qPCR
Atactin-R	CCGATCCAGACACTGTACTTCCTT	qPCR
AtNHX1(AT5G27150)-qF	AGCCTTCAGGGAACCACAAT	qPCR
AtNHX1(AT5G27150)-qR	CTCCAAAGACGGGTCGCATG	qPCR
AtSOS1(AT2G01980)-qF	TTCATCATCCTCACAATGGCTCTAA	qPCR
AtSOS1(AT2G01980)-qR	CCCTCATCAAGCATCTCCCAGTA	qPCR
AtSOS3(AT5G24270)-qF	GAATCCATCGCTCATCAA	qPCR
AtSOS3(AT5G24270)-qR	CCATTTCTTCCTCTTCACA	qPCR
AtRD29a(AT5G52310)-qF	GTGCCGACGGGATTTGACG	qPCR
AtRD29a(AT5G52310)-qR	GTGGTGGTTCCTCTGTTTGATCCAT	qPCR
AtSnRK2.4(AT1G10940)-qF	AACACCCAATCAGCTAACGA	qPCR
AtSnRK2.4(AT1G10940)-qR	GCTCTCCATGAGCTGGTGAT	qPCR
AtNCED3(AT3G14440)-qF	AGACAAATACGCCGAAGA	qPCR
AtNCED3(AT3G14440)-qR	CATACAGGACCCTATCACG	qPCR
